# 음악요법이 초산부의 경막하 무통 분만 중 분만통증, 분만경험, 자아존중감에 미치는 효과: 유사실험 연구

**DOI:** 10.4069/kjwhn.2023.06.21

**Published:** 2023-06-30

**Authors:** Seong Yeon An, Eun Ji Park, Yu Ri Moon, Bo Young Lee, Eunbyul Lee, Dong Yeon Kim, Seong Hee Jeong, Jin Kyung Kim

**Affiliations:** 1 Delivery Room, The Catholic University of Korea, Seoul St. Mary’s Hospital, Seoul, Korea; 1 가톨릭대학교 서울성모병원 분만실; 2 Nursing Innovation Unit, The Catholic University of Korea, Seoul St. Mary’s Hospital, Seoul, Korea; 2 가톨릭대학교 서울성모병원 간호혁신 unit; 3 Obstetrics Ward, The Catholic University of Korea, Seoul St. Mary’s Hospital, Seoul, Korea; 3 가톨릭대학교 서울성모병원 산과병동

**Keywords:** Labor pain, Music therapy, Parturition, Primiparity, Self concept, 분만통증, 음악요법, 분만경험, 초산부, 자아존중감

## Abstract

**Purpose:**

This non-randomized study was performed to evaluate the effects of music therapy on labor pain, the childbirth experience, and self-esteem in women during vaginal delivery.

**Methods:**

In total, 136 primiparous women over 37 weeks of gestation receiving epidural analgesia during vaginal delivery were recruited via convenience sampling. To minimize diffusion effects, data from the control group (n=71) were collected first (April 2020 to March 2021), followed by data from the music group (n=65; April 2021 to May 2022). Participants in the music group listened to classical music during labor, while the control group was offered usual care (no music). Labor pain was measured using a numeric rating scale (NRS), and self-esteem and childbirth experience were collected using self-report questionnaires. Data were analyzed using the independent t-test, chi-square test and Cronbach’s α coefficients.

**Results:**

The overall pain level (NRS) at baseline was 0 in both groups. Mothers in the music therapy group had lower levels of latent pain (t=1.95, *p*=.005), active pain (t=3.69, *p*<.001) and transition-phase pain (t=7.07, *p*<.001) than the control group. A significant difference was observed between the two groups, and the music therapy group expressed more positive perceptions of the childbirth experience (t=-1.36, *p*=.018). For self-esteem, the experimental group’s score was slightly higher, but without a statistically significant difference from the control group.

**Conclusion:**

Using music therapy during labor decreased labor pain and improved the childbirth experience. Music therapy can be clinically recommended as a non-pharmacological, safe, and easy method for nursing care in labor.

Clinical trail number: KCT008561

## Introduction

간호 중재 중 음악요법은 심리적 접근을 이용하여 스트레스를 감소시키는 주요한 중재로 수술 후 통증, 만성 통증 및 암 통증, 분만과 관련하여 불안 및 고통, 혐오감을 감소시키는 데 효과적이며[1-3], 진통제 투여량도 감소시키므로 안전하고 값싼 비약물적 항불안제라 할 수 있다[3-5]. 특히 태교음악은 임부가 아기에게 말을 걸고 음악을 들으면서 임부와 태아에게 안정감과 편안함을 만드는 정서적인 지지를 말하며, 태내 음을 조성하여 임부와 태아의 심리적, 정서적 안정을 도모한다[6]. 태교음악은 임부와 태아와의 상호작용면에서도 태아 애착에 중요한 요소이며[7], 다양한 스트레스 해소법이나 기분전환이 힘든 임부에게 내적인 긴장과 갈등을 해소할 수 있고 정서적 유익을 제공할 수 있다[3,8].

임신과 분만은 여성이 경험하는 발달단계에서 중요한 사건이자 정상적인 생리과정이다[6]. 많은 여성들이 임신과 출산 중에 일어나는 생리적, 심리적 변화에 직면하면서 상당한 스트레스를 받는데[9], 스트레스의 하나인 분만 시의 통증은 분만을 위해서 자궁근육의 수축이 주기적으로 시작될 때부터 태아가 분만될 때까지 산부가 느끼는 주관적인 통증이며, 분만통증 지각 정도에 따라 산부가 긍정적이거나 부정적인 분만경험을 갖게 되고[10], 분만통증의 감소는 산후 우울감을 감소시키고 자신감을 상승시킨다[4].

분만통증 중재 방안으로는 경막외 마취가 보편적으로 사용되어 왔지만 경막외 마취의 시행은 통증을 완벽히 제거할 수 없고, 경막외 마취를 시행하지 않은 산부에 비해 분만통증을 유의하게 감소시키지 않는다는 연구 결과도 있으며[11], 산부에 따라 비약물적 통증 완화를 선호하기도 한다. 따라서 분만 중 통증 완화에 도움을 줄 수 있는 비약물적인 다른 중재법을 함께 시행할 필요가 있다.

분만의 경험은 개인에 따라 다르지만, 초산의 경우는 여성에게 큰 의미를 가지는 경험으로 선명하게 오래도록 기억되고 산부와 가족 간의 관계에 영향을 미치는 중요한 사건이기에 초산부의 분만 경험의 의미를 이해하는 것은 특히 중요하다[12]. 분만을 경험하는 산부는 분만에 대해 긍정적이거나 부정적인 인식을 갖게 되는데, 긍정적인 분만경험은 분만 후 어머니로서 긍정적인 정체감을 형성시켜 모성 역할 수행에 자신감 및 만족도를 증가시킨다[13]. 반면 부정적인 분만경험은 모체에게 생리적, 정신적, 사회적으로 부정적인 경험으로 작용하여 난산, 분만 지연, 분만 후 우울증이나 태아에게 스트레스 등 합병증을 일으키는 변수가 될 수 있다[14].

자아존중감은 자신의 가치에 대한 주관적 평가이고 정신건강의 지표이자 우울을 예측하는 중요한 개인적 요인이다[15]. 산부의 낮은 자아존중감은 주산기 우울증의 주요한 원인으로[16], 높은 자아존중감은 우울을 회복할 수 있는 요인으로 작용하는데 산부의 자아존중감은 스트레스와 가족 지지, 모아 애착과 신체상에 상관성이 높다[17,18].

산부를 대상으로 간호중재 프로그램의 효과를 검정한 선행연구들은 산부에게 라마즈 분만법과 함께 음악요법을 같이 적용하였을 때 통증 감소, 분만시간 단축, 분만 자신감 증가와 불안감 감소로 분만경험을 긍정적으로 인식한다는 연구도 있었다[13,19]. 또한 배우자의 손마사지가 무통분만 초산부의 불안을 낮추고 출산경험 지각에 영향을 미치며, 듀라터치 분만 교육이 분만 자신감에 유의미한 영향을 미친다는 연구 결과와[20,21] 삼음교 지압이 분만통증을 감소시켰다는 결과도 있었다[22].

음악요법이 조기진통 임부의 조기진통 스트레스를 감소시킨다는 연구도 있었고[5], 특히 분만과정에서 산부에게 음악을 들려준 후 산부의 주관적 긴장감과 통증강도가 감소하고 불안과 스트레스와 관련된 호르몬 변화와 근육 이완을 유도하여 출산을 도와준다고 하는 연구들이[4,23] 있었다. 음악요법이 출산 중 고통과 불안을 줄이고 다각적인 심리적 지원을 제공할 수 있어 여성들에게 중요한 역할을 한다는 연구[24]를 볼 때 음악요법은 분만통증 감소와 자아존중감 상승에 도움을 준다고 생각된다. 음악요법의 가치는 산과학을 비롯한 다양한 임상 분야에서 간호사가 계속 실현해 왔는데[25], 국내에서는 분만 진행과정 중 음악요법이 초산부에게 어떠한 영향을 끼치는지에 관한 연구는 거의 이루어지지 않고 있는 실정이다. 이에 본 연구는 처음 분만을 경험하는 산부에게 진통 중 음악요법을 적용하여 분만통증, 분만경험, 자아존중감에 대한 효과를 규명하고 궁극적으로 초산부가 긍정적인 분만경험을 할 수 있도록 하고자 시도하였다.

본 연구의 목적은 진통 중 음악요법 간호중재가 초산부의 분만에 미치는 영향을 알아보고자 하며, 구체적인 목적은 다음과 같다.

1) 진통 중 음악요법 중재군과 대조군의 분만통증 차이를 확인한다.

2) 진통 중 음악요법 중재군과 대조군의 분만경험 차이를 확인한다.

3) 진통 중 음악요법 중재군과 대조군의 자아존중감 차이를 확인한다.

## Methods

Ethics statement: This study was approved by the Institutional Review Board of The Catholic University of Korea (KC19QESI0637). Informed written consent was obtained from the participants.

### 연구 설계

**Figure 1.**
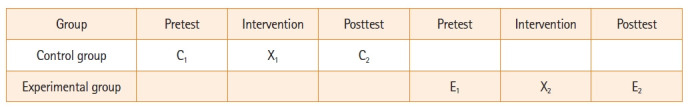
The study design. C_1_, E_1_: General characteristics, self-esteem; C_2_, E_2_: labor pain, childbirth experience, self-esteem; X_1_: usual care (hospitalization manual, epidural labor analgesia, spouse’s participation in delivery); X_2_: usual care, music therapy during labor.

본 연구는 진통 중 음악요법을 받은 초산부 중재군과 대조군의 분만통증, 분만경험, 자아존중감을 비교하기 위한 비동등성 대조군 전후 시차설계 유사실험 연구로(Figure 1), TREND (Transparent Reporting of Evaluations with Nonrandomized Designs) guidelines에 따라 기술하였다[26].

### 연구 대상

**Figure 2.**
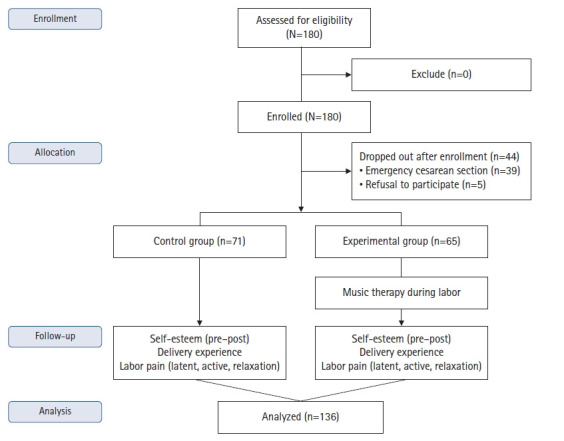
CONSORT flow diagram modified for a non-randomized trial design.

2020년 4월 20일부터 2022년 5월 11일까지 2년에 걸쳐 서울에 소재한 가톨릭대학교 서울성모병원 분만실에 입원 중인 산부를 대상으로 하였다. 대상자 선정기준은 37주 이상의 자연분만을 하는 초산부 중 경막하 무통분만으로 분만하며 배우자가 분만에 참여하는 산부였으며, 37주 미만의 조산을 하거나 응급 분만으로 제왕절개하는 산부는 제외하였다. 표본의 크기는 G*power 3.1.9.4를 이용하여 독립 t검정으로 분석할 때 유의수준 .05, 효과크기 .50 [8], 검정력 .80으로 하여 산출하였다. 연구 대상자는 중재군 64명, 대조군 64명으로 총 128명이며, 산부의 특성상 분만과정 중 참여가 어렵거나 제왕절개 분만으로 변경될 가능성이 높아 탈락률을 30%로 고려하여 각각 90명 총 180명이 필요하였다. 자료 수집 중 태아곤란증(fetal distress)이나 태아 하강 부전으로 인한 응급 제왕절개로 39명이 탈락하였고, 연구 도중 산부가 연구 참여를 원치 않아 참여를 중단한 5명을 제외하고 136명의 자료를 수집하였다. 대조군은 71명, 음악요법 간호중재를 받은 중재군은 65명이었다(Figure 2).

### 연구 도구

#### 분만통증

분만통증의 측정은 Farrar 등[27]의 민감도 .73과 특이도 .75의 신뢰성과 타당성이 있는 도구인 수치평가척도(numeric rating scale, NRS)로 측정하였다. 주관적인 측정방법으로 널리 사용되는 NRS는 0점 ‘전혀 통증이 없다’에서 10점 ‘매우 통증이 심하다’의 11포인트 척도이며, 환자가 자신의 통증을 정의된 척도로 평가하는 것으로 숫자가 높을수록 분만통증이 심한 것을 의미한다. 분만통증을 측정한 시기는 분만1기인 잠재기(자궁경관 개대 2–3 cm 진행된 상태), 활동기(자궁경관 개대 4–7 cm 진행된 상태), 이행기(자궁경관 개대 8–10 cm 진행된 상태)에 각각 한 번씩 NRS 점수로 질문하여 산부의 주관적인 통증을 세 번 측정하였다.

#### 분만경험

분만경험 측정도구는 원 저자인 Marut과 Mercer [28]가 산부의 분만경험을 측정하기 위해 개발한 척도(Perception of Birth Scale)를 Kim [29]이 번안한 도구를 이메일을 통해 도구 사용 승인을 받고 본 연구에 맞게 제왕절개 부분을 삭제하고 자연분만으로 문장을 수정하여 20가지 문항으로 구성하였다. 임상 경력 10년 이상의 석사 이상 산부인과 수간호사 5명, 산부인과 교수 2명으로 구성된 7인의 전문가 집단이 내용타당도(content validity index, CVI) .86 이상인 20문항을 추출하였고, 평균 CVI값은 .98이었다. 문항의 구성은 4점(‘매우 그렇지 않다’ 1점에서 ‘매우 그렇다’ 4점)으로 합산하며(가능 점수 범위, 20–80점) 점수가 높을수록 분만경험이 긍정적임을 의미한다. 선행연구[29]에서 신뢰도 Cronbach’s α값은 .80이었고 본 연구에서 도구의 신뢰도는 Cronbach’s α .86이었다.

#### 자아존중감

자아존중감 측정도구는 Rosenberg [30]가 개발한 Rosenberg Self-Esteem Scale (RSES) 검사를 Kwon [31]이 번안한 것을 이메일을 통해 도구 사용 승인을 받고 사용하였다. 10문항으로 구성된 도구는 자아존중감을 4점 척도(‘매우 그렇지 않다’ 1점에서 ‘매우 그렇다’ 4점)로 합산하며(가능 점수 범위, 10-40점) 점수가 높을수록 자아존중감이 높음을 의미한다. 선행연구[31]에서 신뢰도 Cronbach’s α값은 .90이었고 본 연구에서 도구의 신뢰도 Cronbach’s α값은 .73이었다.

#### 일반적 특성

일반적 특성은 연령, 병실 종류, 재태연령, 회음부 열상 유무, 태교음악 경험 유무, 1분 아프가(Apgar) 점수, 5분 아프가 점수, 신생아 체중, 신생아 중환자실 입실 유무를 조사하였다.

### 연구 절차

서울에 소재한 가톨릭대학교 서울성모병원 분만실 게시판에 본 연구에 대해 게시하여 대상자 모집 공고를 하고, 스스로 참여를 원하는 산부에게 연구의 목적과 방법을 공동 연구자가 설명하여 동의를 받았다. 실험의 확산을 방지하고자 대조군은 2020년 4월 20일부터 2021년 3월 5일까지 자료 수집을 하였고 시차설계를 적용하여 실험군은 1개월 뒤인 2021년 4월 3일부터 2022년 5월 12일까지 자료 수집을 하였다. 중재군과 대조군 모두 동의서 작성 후 일반적인 특성을 조사하고 자아존중감 설문을 1차 시행한 후, 분만과정에 걸쳐 각각 NRS 점수로 질문하여 산부의 주관적인 통증을 세 번 측정하였다.

본 연구에서 대조군과 중재군 모두 배우자가 분만에 참여하여 제대결찰을 하였으며, 대조군은 음악요법을 중재하지 않았고, 중재군은 클래식이 산부에게 안정감과 편안함을 주는 것에 근거하여[32] 모짜르트, 슈베르트의 클래식 음악으로 구성된 태교음반 CD 총 48곡을 분만실에서 동일한 CD 플레이어를 통해 동일한 CD로 산부가 원하는 소리 크기로 제공하였다. 공동 연구자가 음악요법의 중재자로, 산부의 자궁 수축이 시작되는 분만 1기부터 태아가 만출되는 분만 3기 끝까지 평균 8–10시간 음악요법을 제공하였다. 자연분만 후 4시간 이내에 자아존중감 10문항 2차 설문지와 분만경험 20문항 설문지를 측정하였다. 사후 측정 및 분만이 완료된 후 두 군 모두에게 사례품(신생아용 유기농 손수건)을 제공하였다.

### 자료 분석 방법

수집된 자료는 IBM SPSS ver. 24.0 (IBM Corp., Armonk, NY, USA)을 이용하여 분석하였으며, 도구의 신뢰도는 Cronbach’s α 계수로, 대상자의 일반적 특성은 Shapiro-Wilk test를 시행하여 각각의 정규성 분포를 확인한 후 빈도, 백분율, 평균, 표준편차 등 서술적 통계 자료를 이용하여 분석하였다. 중재군과 대조군 간의 일반적 특성은 카이제곱 검정과 독립 t검정을 이용하여 동질성 검사를 하였다. 중재군과 대조군 간의 음악요법 후 분만통증, 분만경험은 독립 t검정으로 분석하였다. 음악요법 후 자아존중감은 전후 비교는 대응표본 t검정(paired t-test)으로, 중재군과 대조군 간의 차이 비교는 독립 t검정으로 분석하였다.

## Results

### 대상자의 일반적인 특성

연구에 참여한 산부 136명의 평균 연령은 32.7±3.3세로, 30–34세가 83명(61.0%)로 가장 많았고 35세 이상이 32명(23.5%)이었다. 진통 및 분만장소는 다인실에서 진통하다 분만장으로 옮겨서 분만한 산부가 75명(55.1%), labor, delivery, recovery (LDR) 병실에서 진통과 분만을 함께 한 산부는 61명(44.9%)이었다. 재태 연령은 39주가 60명(44.1%)으로 가장 많았으며 평균 39.25±0.86주였다. 분만으로 인한 회음부 열상은 9명(6.6%)이었고, 분만 전 99명(72.8%)이 태교음악의 경험이 있다고 응답하였다. 신생아의 1분 아프가 평균 점수는 8.58±1.13점, 5분 아프가 평균 점수는 9.65±0.84점이었다. 신생아의 체중은 2.5–3.4 kg이 112명(82.4%)으로 가장 많았고 평균 3.1±0.3 kg이었다. 신생아가 중환자실로 이송된 경우는 15명(11.0%)이었으며 중재군과 대조군의 일반적 특성을 비교한 결과 두 군은 유의한 차이를 보이지 않아 동질성을 확인하였다(Table 1).

### 음악요법이 분만통증과 분만경험에 미치는 영향

실험군과 대조군 산부는 유도분만을 위해 입원했으며 입원 시 분만 통증이 없는 상태였다. 진통 중 음악요법이 초산부의 분만통증에 미치는 영향을 비교하였을 때, 분만통증은 잠재기에서 대조군은 3.65±1.79점, 중재군은 3.03±1.90점으로 중재군의 분만통증이 대조군보다 점수가 낮았다(t=1.95, *p*=.005). 활동기에서의 분만통증은 대조군은 3.83±2.03점, 중재군은 2.71±1.53점으로 중재군의 분만통증이 대조군보다 유의하게 낮았다(t=3.69, *p*<.001). 이완기에서의 분만통증은 대조군 5.10±2.14점, 중재군 2.88±1.49점으로 중재군이 유의하게 낮았다(t=7.07, *p*<.001). 진통 중 음악요법 중재 후 분만경험은 평균 58.97±7.72점이었으며 대조군 58.11 ±6.94점, 중재군 59.91 ±8.45점으로 유의한 차이를 보여 중재군이 긍정적으로 인지함을 확인하였다(t=–1.36, *p*=.018) (Table 2).

### 음악요법이 자아존중감에 미치는 영향

초산부의 자연분만 시 음악요법이 자아존중감에 미치는 영향을 비교하였을 때, 중재 전 대조군은 32.34±3.22점, 중재군은 32.43±3.01점으로 두 군은 동질하였다. 중재 처치 후 대조군은 32.34±3.22점에서 32.23±3.37점으로 0.11점 감소하고, 중재군은 32.43±3.01점에서 32.63점으로 0.20점 증가하였으나, 중재 전후 차이를 비교하면 두 군 간에 유의한 차이가 없었다(Table 3).

## Discussion

본 연구에서 음악요법은 분만통증을 감소하는 데 잠재기, 활동기, 이완기 모두 효과적이었다. 선행연구에서 자연분만 산부의 회음부 절개 봉합 시 신생아와의 피부접촉군(캥거루 케어), 음악요법군, 대조군의 세 군을 대상으로 분만통증과 불안에 미치는 영향을 비교한 연구에서는 음악요법군이 가장 통증 점수가 낮아 음악요법의 통증 조절 효과를 입증한 연구도 있었다[33]. 자연분만 산부를 대상으로 출산과정 중 음악요법을 중재한 연구[4]에서 산후 불안감과 통증이 감소하고 출산 만족도가 높아지며 초기 산후 우울증 발생률이 감소하였으며, 자연분만 중 자유로운 자세와 음악요법을 함께 중재한 연구[34]에서도 산부의 분만통증이 전통적인 분만을 진행한 대조군보다 유의한 감소를 보였다. 이처럼 스트레스를 감소시키는 간호중재 중 음악요법은 관심 전환 방법 중 하나로 심리적 접근을 이용하는 주요한 간호중재이며 이완요법[8]이라고 볼 수 있어 분만통증에 효과가 있는 것으로 보인다.

선행연구에서 산부의 분만 후 음악요법이 산후 우울과 모아 애착에 긍정적인 효과를 보였고[35], 정신적, 육체적, 음악적 측면에서 몸을 움직이며 음악을 듣는 홀리스틱 유리드믹스 음악교육도 임부의 불안과 우울, 신체적 불편감 완화에 긍정적인 영향을 미쳤다[36]. 본 연구에서 음악요법도 신체적 불편감인 통증을 완화하는 것뿐만 아니라 긍정적인 분만경험에 좋은 영향을 미쳐 음악요법이 지속될 필요가 있다고 본다. 이처럼 음악요법은 긍정적인 분만경험을 보였는데, 음악요법과 긍정적인 분만경험에 대한 연구는 미비하여 다른 연구와 비교하기에는 한계가 있다.

본 연구에서 음악요법 중재 전후 차이를 비교하면 자아존중감에서 유의한 차이가 없었는데, 임신 7–9개월의 미혼모 임부를 대상으로 음악 감상, 악기 연주와 같은 음악활동 프로그램이 자아존중감에 미치는 영향을 분석한 연구[37]에서 중재군의 자아존중감이 증가하였던 결과와 달랐다. 이는 본 연구의 대상자들은 미혼모와 특성과 달리 배우자의 지지를 받는 산부였다는 점에서 차이가 있는 것으로 생각된다.

본 연구는 단일 기관의 분만실만을 대상으로 조사하여 산부의 지역적인 분포가 고르지 못하므로 연구 결과를 일반화하는 데에는 제한이 있다. 그러나 분만이 처음이라 불안과 두려움을 가진 초산부에게 음악요법 간호중재가 심리적 안정과 지지간호를 제공함으로써 분만통증에도 효과적이고 분만경험에도 긍정적인 효과를 보였으므로, 임상현장에서 적극적으로 반영하기를 기대한다. 음악요법 간호중재가 자아존중감과 분만경험에 미치는 영향에 대한 추가적인 반복 연구나 고위험 신생아를 분만하는 산부를 대상으로 한 추가 연구도 제언한다.

본 연구는 초산부의 자연분만 시 진통 중 음악요법 간호중재가 분만통증, 자아존중감, 분만경험에 미치는 영향을 조사하였다. 음악요법은 분만 잠재기, 활동기와 이완기에서 모두 유의하게 분만통증을 감소시켰고, 분만경험에도 긍정적인 영향을 미쳤다. 자아존중감에는 유의한 차이가 없었지만, 손쉽게 제공할 수 있는 지지간호의 한 영역으로 특히 처음 분만을 경험하며 많은 불안을 경험할 수 있는 산부에게 적절했다. 간호실무 측면에서 음악요법을 제공하여 분만통증을 완화하고 긍정적인 분만경험을 통해 모아 건강에 기여하는 근거를 마련하였다는 데 본 연구의 간호학적 의의가 있다.

## Figures and Tables

**Table 1.** General characteristics according to whether primiparous women received prenatal music therapy during labor (N=136)

Characteristic	Categories	n (%) or mean±SD			χ^2^ or t	*p*
		Total (n=136)	Experimental group (n=65)	Control group (n=71)		
Age (year)		32.7±3.3	32.5±3.3	32.9±3.2	0.73	.469
	<30	21 (15.5)	13 (20.0)	8 (11.3)	2.02	.364
	30-34	83 (61.0)	38 (58.5)	45 (63.4)		
	≥35	32 (23.5)	14 (21.5)	18 (25.3)		
Labor room	LDR room	61 (44.9)	32 (49.2)	29 (40.8)	0.97	.326
	Multi-bed room	75 (55.1)	33 (50.8)	42 (59.2)		
Gestational age (week)		39.25±0.86	39.36±0.85	39.15±0.87	1.41	.161
	≤38	44 (32.4)	21 (32.3)	23 (32.4)	1.40	.498
	39	60 (44.1)	26 (40.0)	34 (47.9)		
	≥40	32 (23.5)	18 (27.7)	14 (19.7)		
Perineal laceration	Yes	9 (6.6)	6 (9.2)	3 (4.2)		.310^†^
	None	127 (93.4)	59 (90.8)	68 (95.8)		
Prenatal music experience	Yes	99 (72.8)	49 (75.4)	50 (70.4)	0.42	.516
	None	37 (27.2)	16 (24.6)	21 (29.6)		
Apgar score, 1 minute		8.58±1.13	8.78±0.45	8.39±1.48	1.10	.268
	≤8	32 (23.5)	13 (20.0)	19 (26.8)	0.86	.353
	≥9	104 (76.5)	52 (80.0)	52 (73.2)		
Apgar score, 5 minute		9.65±0.84	9.82±0.43	9.51±1.07	1.67	.096
	≤8	6 (4.4)	1 (1.5)	5 (7.0)		.211^†^
	≥9	130 (95.6)	64 (98.5)	66 (93.0)		
Weight of infant (kg)		3.1±0.3	3.1±0.3	3.1±0.3	1.22	.221
	<2.5	4 (2.9)	3 (4.6)	1 (1.4)		.560^†^
	2.5–3.4	112 (82.4)	52 (80.0)	60 (84.5)		
	≥3.5	20 (14.7)	10 (15.4)	10 (14.1)		
Admission to NICU	Yes	15 (11.0)	5 (7.7)	10 (14.1)	1.41	.235
	None	121 (89.0)	60 (92.3)	61 (85.9)		

LDR: Labor, delivery, recovery room; NICU: neonatal intensive care unit.

† Fisher’s exact test.

**Table 2.** Effects of prenatal music care on labor pain and childbirth experience during primiparous women’s labor (N=136)

Characteristic	Mean±SD			t	*p*
	Total (n=136)	Experimental group (n=65)	Control group (n=71)		
Labor pain					
Latent phase	3.35±1.86	3.03±1.90	3.65±1.79	1.95	.005
Active phase	3.29±1.89	2.71±1.53	3.83±2.03	3.69	<.001
Transition phase	4.04±2.16	2.88±1.49	5.10±2.14	7.07	<.001
Childbirth experience					
Post-intervention	58.97 ±7.72	59.91 ±8.45	58.11 ±6.94	–1.36	.018

**Table 3.** Effect of prenatal music care on self-esteem during primiparous women’s labor (N=136)

Variable	Mean±SD		t	*p*
	Experimental group (n=65)	Control group (n=71)		
Self esteem				
Preintervention	32.43±3.01	32.34±3.22	0.17	.863
Postintervention	32.63±3.52	32.23±3.37	0.69	.494
Post–Pre	0.20±2.43	–0.11±2.35	0.18	.858
